# Mechanism of the Exchange Reaction in HRAS from Multiscale Modeling

**DOI:** 10.1371/journal.pone.0108846

**Published:** 2014-10-01

**Authors:** Abhijeet Kapoor, Alex Travesset

**Affiliations:** Physics and Astronomy, Iowa State University, Ames, Iowa, United States of America; Beatson Institute for Cancer Research Glasgow, United Kingdom

## Abstract

HRAS regulates cell growth promoting signaling processes by cycling between active (GTP-bound) and inactive (GDP-bound) states. Understanding the transition mechanism is central for the design of small molecules to inhibit the formation of RAS-driven tumors. Using a multiscale approach involving coarse-grained (CG) simulations, all-atom classical molecular dynamics (CMD; total of 3.02 *µs*), and steered molecular dynamics (SMD) in combination with Principal Component Analysis (PCA), we identified the structural features that determine the nucleotide (GDP) exchange reaction. We show that weakening the coupling between the SwitchI (residues 25–40) and SwitchII (residues 59–75) accelerates the opening of SwitchI; however, an open conformation of SwitchI is unstable in the absence of guanine nucleotide exchange factors (GEFs) and rises up towards the bound nucleotide to close the nucleotide pocket. Both I21 and Y32, play a crucial role in SwitchI transition. We show that an open SwitchI conformation is not necessary for GDP destabilization but is required for GDP/Mg escape from the HRAS. Further, we present the first simulation study showing displacement of GDP/Mg away from the nucleotide pocket. Both SwitchI and SwitchII, delays the escape of displaced GDP/Mg in the absence of GEF. Based on these results, a model for the mechanism of GEF in accelerating the exchange process is hypothesized.

## Introduction

HRAS, a member of the Ras superfamily, regulates cell growth promoting signaling processes by cycling between active (GTP-bound) and inactive (GDP-bound) states [1], [2]. The active state is recognized by downstream effectors to pass on the signal. The intrinsic low rates of interconversion between the two states is enhanced by interactions with two regulatory proteins. While guanine nucleotide exchange factors (GEFs) catalyze the exchange of GDP to GTP, the inactive state is regenerated by hydrolysis of GTP to GDP, accelerated by a GTPase activating protein (GAP) [3]. Mutations intervening normal Ras functioning are associated with several human cancers and developmental disorders [4], [5]. Oncogenic Ras proteins, found in more than 20% of human tumors, are insensitive to GAP action, leading to a permanent active state, which results in uncontrolled cell growth.

The dynamic nature of the SwitchI (residues 25–40) and SwitchII (residues 59–75) regions in HRAS structure and their participation in regulation of inactive/active state transitions has been long established [1], [6]–[15]. Structure-based mutagenesis studies established the specific role of SwitchII in anchoring the GEF and SwitchI in GDP dissociation [12]. As seen in the HRAS-GEF crystal complex, GEF opens up the nucleotide binding pocket and forms an extensive interface with the two switch regions, distorting their conformation such that it no longer favors the nucleotide binding [7]. Experiments have shown that a transient ternary complex (HRAS-GDP-GEF) precedes the formation of the binary complex [16]. Interestingly, similar structural rearrangements as seen in the HRAS-GEF complex were also observed in the ternary complex of a plant G protein Rop4 in complex with GDP and its GEF [17].

Essential features of the GEF-catalyzed exchange reaction have remained mostly uncharacterized due to the difficulty in crystalizing the intermediate structures. The interconversion between the GDP-GTP bound form, in the absence of regulatory proteins, has been studied computationally using targeted molecular dynamics [13] or accelerated molecular dynamics [14], and also through classical molecular dynamics (CMD) of HRAS-G12V [18]. Further insight into the activation mechanism was provided by Kobayashi and Saito [15] based on the simulation of HRAS-GTP with SwitchI in state 1 (weak T35-Mg coordination) who proposed a reaction path in which the HRAS-GEF complex is converted to the active protein via state 1. Recently, a combination of steered molecular dynamics (SMD) and CMD was used to study the activation mechanism by replacing the GDP with GTP in the inactive structure [19]. Yet, several fundamental questions such as what structural features control intrinsic reaction rates, how the presence of GEF catalyzes these reactions, or the allosteric changes involved to name a few, remain unanswered.

In this paper, we use a multiscale approach including coarse-grained (CG) dynamics [20], [21], all atom molecular dynamics (MD), SMD, and techniques such as Principal Component Analysis (PCA) to characterize the conformations of HRAS. GDP- and GTP-bound conformations of HRAS were first simulated using the CG model. Dynamics resulting from CG simulations were then used as guide to set-up all atom MD simulations. The goal is to understand the specific structural features involved in the regulation of the intrinsic exchange reaction, namely GDP exchange, providing an understanding of the ways in which the process would be catalyzed by GEF. Such knowledge is also critical to provide a framework for design of small molecule ligands and prevent the formation of the permanent active state by inhibiting exchange activity in oncogenic mutants.

## Materials and Methods

### Simulation Setup and Analysis

HRAS simulations, both CG and all-atom, were performed in GDP- (PDB id: 4Q21) and GTP-bound (PDB id: 5P21 and 3RSO) states. A CG representation of the protein as described in [20], [21], wherein the backbone is represented with atomic resolution but the sidechain with single bead, was used to model HRAS. The model has sufficient predictive power so that starting from random initial conformations it properly folded 19 proteins into their native states, and provides a reliable description of the dynamics as compared with all atom simulations [20], [21]. CG mapping of GDP/GTP is described in Text S1. All CG simulations were run on the HOOMD-blue package [22] running on Graphics Processing Units (GPUs). CG simulations used brownian dynamics within the canonical ensemble and were performed for 100 million time-steps.

All-atom simulations were performed using program NAMD [23] and CHARMM27 force field for proteins. Simulations were performed using periodic boundary conditions, TIP3P water and the system was neutralized by adding counter ions. A 2fs time-step was used with a 12 Å cut-off for VDW interactions and full particle-mesh Ewald electrostatics. All simulations started by first minimizing the structure followed by constant volume heating for 10ps. This was followed by a constant temperature and constant pressure (1atm) dynamics for 100ns or more including a 1ns equilibration (Table S1 provides details). Multiple runs were started from the same initial configuration but using different seed.

Trajectory visualization and figure preparation was done using VMD [24]. Prior to structure analysis, the crystal structures and trajectories were aligned using the backbone atoms N, CA, and C of core residues as defined in [18]. The time evolution of the root mean square deviation (RMSD) from the respective initial structures and the root mean square fluctuations (RMSF) of C*_α_* atoms was used to assess the stability of HRAS in different simulations. Cross-correlation analysis for all the C*_α_* atom pairs was performed to identify residues with correlated motions. The cross-correlation coefficient, C*_ij_*, for atom pairs, i and j, is defined as: 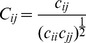
(1)where c*_ij_* is the corresponding covariance matrix element, given as:
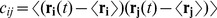
(2)where **r** *_i_* (t) is the coordinate of atom i at time t and 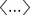 denotes time averages.

PCA was applied to examine the relationships between different structures and to determine the conformational space sampled by various simulations. In this study, PCA analysis was performed using C*_α_* atom coordinates. In PCA, the diagonalization of covariance matrix is performed to obtain the principal components (PC), which are the eigenvectors of the covariance matrix. The inter-conformer relationships can be studied by projecting the structures onto the sub-space defined by the largest principal components.

Contact analysis was performed to determine the common contacts involved in SwitchI transition from open to closed states. The details of contact identification are given in Text S1.

## Results

### Experimental structures can be clustered into three major groups

**Figure 1:**
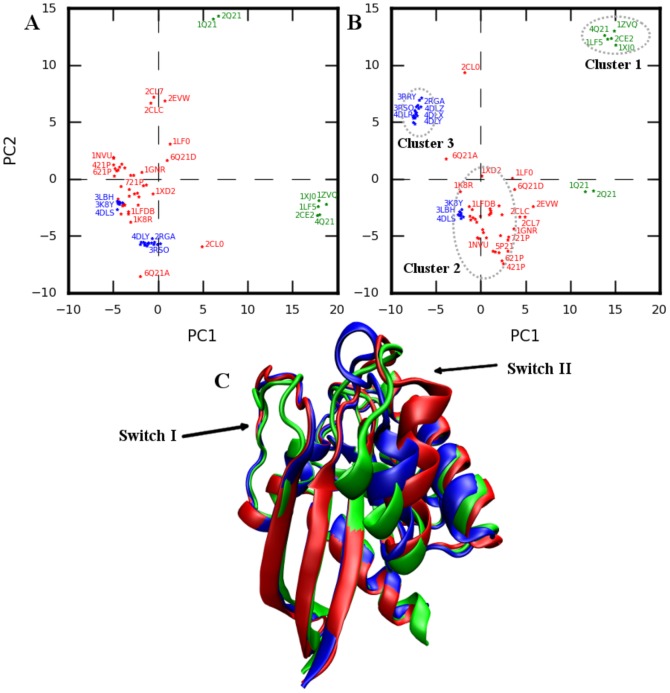
PCA analysis of experimental structures. Projection of structures onto the first two PCs obtained using A) 46 structure dataset and B) 71 structure dataset. Green: HRAS-GDP bound; Red, Blue: HRAS-GTP bound conformations. Experimental structures groups in to three major clusters highlighted in B. C. Representative structures from the three clusters, 4Q21 (green), 3RSO (blue), and 5P21 (red). An enlarged version of Figure 1A-B is also shown in Figure S1 highlighting clearly the PDB ids of most of the structures used for analysis.

In the PCA analysis of 46 RAS structures by Gorfe et al. [18], two distinct clusters were identified along principal component (PC) PC1 corresponding to the nature of bound nucleotide. Adding 25 new recently solved structures (blue stars Figure 1A-B, Figure S1 and Table S2) [25]–[28] to the 46 structure dataset in Ref [18] and projecting the conformations onto the sub-space defined by the first two PCs obtained from the 46 structure dataset (Figure 1A) again identifies two clusters along PC1 corresponding to GTP (blue and red stars in Figure 1A) and GDP bound (green stars) structures. Interestingly, moving along PC2, the group formed by GTP-bound conformations can now be further sub-divided into two clusters. Overall, the three clusters become much more evident when the structures are projected on to the sub-space defined by the first two PCs obtained using the 71 structure dataset (Figure 1B). The GTP-bound structures in cluster 2 and cluster 3 mainly differ in the orientation of SwitchII-helix (residues 66 to 75, 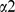), SwitchII loop (residues 59 to 66, L4), loop7 (residues 105–109, L7), and C-terminal part of 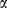
3 (residues 87–104). 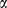
2 in GTP-bound structures (cluster 3) occupies an intermediate position compared to the one observed in 4Q21 (cluster 1) and 5P21 (cluster 2). Based on the PCA analysis, we selected one representative structure from each cluster (4Q21, 5P21, and 3RSO) for further investigation using MD (Figure 1C).

### Equilibrium dynamics of HRAS from CG simulations are in good agreement with all-atom simulations

Dynamics resulting from the CG simulations of the three representative structures were compared with all-atom simulations (performed at 300K). Detailed analysis assessing the stability, conformational space sampled, and the correlations arising from the CG and all-atom simulations is given in Text S1 and Figure S2, S3, and S4. Fluctuations from the CG simulations are observed to be in good-agreement with those of the all-atom simulations. In addition, enhanced sampling is observed in CG simulations (see Text S1).

### SwitchI/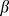
2 semi-opening is a thermally activated process coupled to SwitchII

CG simulations of HRAS at different temperatures showed that the opening of SwitchI and partial melting of 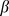
2 strand (residues 38–46; semi-open conformation) is intrinsic to HRAS motion (occurs in both GDP and GTP bound state) at higher temperature and, rather remarkably, does not require GEF (data not shown). To validate this result, we performed all-atom MD for each of the representative structures at different temperatures (300K, 360K, and 400K). Overall the three structures remained stable at high temperatures with increased fluctuations limited to parts of SwitchI, SwitchII, 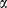
3, L7, and interswitch regions (Figure S5). In agreement with the results from CG model, semi-open conformations of SwitchI/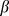
2 were observed in all atom simulations at higher temperature (irrespective of the bound nucleotide; Figure S5).

**Figure 2:**
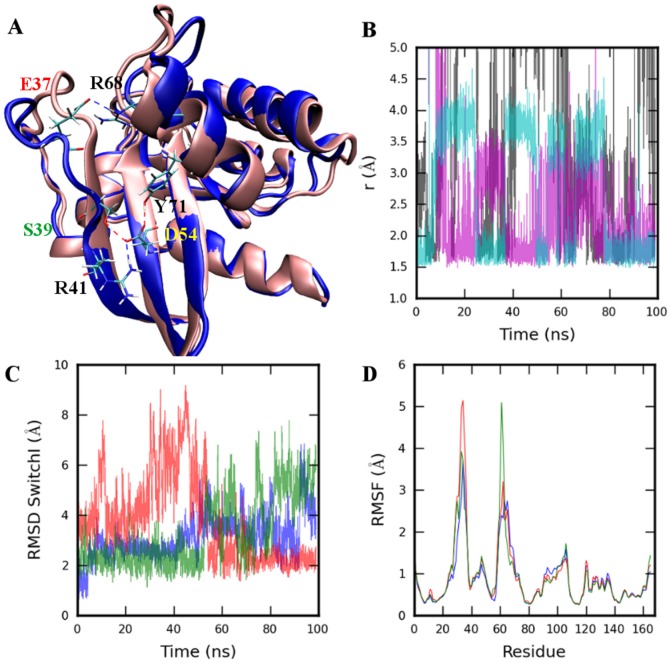
Wild type and mutant 4Q21 all-atom simulations. A. Coupling between the SwitchI and SwitchII regions as a result of direct hydrogen bond (shown as dotted red and blue lines) formation between residues E37 and R68, and the network of hydrogen bonds formed between S39, R41, D54, and Y71 showing D54 mediated coupling. Simulation was performed at 300K temperature. B. Distance between the hydrogen bond donor/acceptor atoms for residue pairs E37-R68 (black), S39-D54 (magenta), and Y71-D54 (cyan) for 4Q21 simulation at 300K C. RMSD of SwitchI (residues 25–40) and D. C*_α_*-RMSF (root mean square fluctuation) from 99ns simulations of 4Q21 (blue), 4Q21E37A (red), and 4Q21S39A (green) at temperature 360K. Structure alignment was performed using backbone atoms N, CA, and C of core residues as defined in [18].

Comparison of 4Q21 simulations at different temperatures shows a weak coupling between SwitchI and SwitchII that appears critical for SwitchI opening. Coupling here refers to the link between the two switch regions observed as a result of the residue interactions. The simulation at 300K shows a coupling between the two regions through direct hydrogen bond formations between residues E37 and R68 and a D54 mediated coupling as a result of a network of hydrogen bonds formed by residues S39, R41, D54, and Y71 (Figure 2A, B). However, at higher temperatures, fluctuations in both 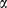
2 and Y71 sidechain become higher, and as a result the coupling mediated by D54 weakens. Also, a loss of interaction between E37 and R68 is observed, which results in only transient interactions of E37 with other SwitchII residues (S65 and Y71), eventually leading to SwitchI opening. Further weakening the coupling by E37A and S39A mutations (4Q21E37A and 4Q21S39A simulations) resulted in increased fluctuations of SwitchI (Figure 2C-D). As evident from the RMSD of SwitchI (Figure 2C), the opening is faster, is of considerably longer duration, and occurs early in the simulation compared to wild type 4Q21. The effect of these mutations, especially S39A mutation, becomes more apparent on comparing the distribution of distances between the SwitchI residues and representative GDP atoms. While in 4Q21 higher fluctuations were mainly observed in the C-terminal part of SwitchI (residues 32 and above), in mutant simulations, increased fluctuations are also observed in the N-terminal part of SwitchI (Figure S6A, S7). Interestingly, in the 4Q21S39A simulation, a change in the orientation of Y32 sidechain was observed, which allowed it to form hydrogen bonds with oxygen atoms of P*_α_* group in GDP preventing further opening of SwitchI (Figure S6B).

Contrary to the inactive state, in the crystal structure of the active state (5P21), both R68 and Y71 have different orientations and do not interact with E37 and D54. No interaction among these residues is observed in the simulations at 300K as well. In fact, hydrogen bond interactions between T35 and D57 and transient interaction between Q61 and backbone of T35 are observed. Unlike 4Q21, SwitchI opening was not observed at 360K and only partial opening was observed at 400K (Figure S5B). At 400K, T35-Mg coordination weakens after about 12ns, and the hydrogen bond interaction with D57 is also lost. This resulted in SwitchI opening. Interestingly, as SwitchI opened, 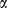
2 changed its orientation to the one observed in 4Q21 which resulted in Y71 forming back the network of hydrogen bond between residues S39, R41, D54, and Y71.

In the case of 3RSO, both the 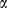
2 and the residues Y71 and R68 are positioned somewhat intermediate to its position in 4Q21 and 5P21. Within the 3RSO simulation at 300K, like 5P21, no interaction between Y71 and D54 was observed but, analogously as in 4Q21, E37 interacted frequently with R68. E37 also formed hydrogen bond interactions with T58 and Y71 after it loses contact with R68. SwitchI/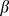
2 opening in 3RSO was observed at 360K. Early in the simulation the coordination between T35 and Mg weakens, this is followed by the loss of hydrogen bond interaction between T35-D57 and E37-Y71.

### Open SwitchI conformation transitions to its closed state

We performed simulations of 4Q21 (GDP-bound) with its SwitchI modeled in an open conformation as observed in the HRAS-GEF complex [7] keeping the rest of the structure the same as 4Q21 (hereafter this structure will be referred to as the 4Q21-OpenSI). CG simulations of 4Q21-OpenSI showed that the open conformation of SwitchI resulted in destabilization of GDP, eventually displacing GDP/Mg from the nucleotide pocket. Destabilization refers to the state in which both the base and the ribose group lose contact with HRAS and GDP remains anchored via phosphate/Mg-HRAS interactions only. Results from one such CG trajectory are shown in Figure S8. Interestingly, the N-terminal part of SwitchI rises up towards GDP while the helical turn (formed by residues 37–39) in the C-terminal part of SwitchI remained intact throughout the simulation (blue conformation in Figure S8A) preventing the complete transition of SwitchI towards its closed state (seen in 4Q21). Early in the simulation sidechain hydrogen bond forms between residue pairs D30-R149 (blue in Figure S8B), H27-E153 (red), and T20-T35 (green) which are replaced, at around 26 million timesteps, by D30-K147 (black) and the sidechain-mainchain hydrogen bond S17-Y32 (magenta), resulting in partial transition of SwitchI towards its closed state as also indicated by the RMSD of SwitchI residues with its conformation in 4Q21 (Figure S8C).

**Figure 3:**
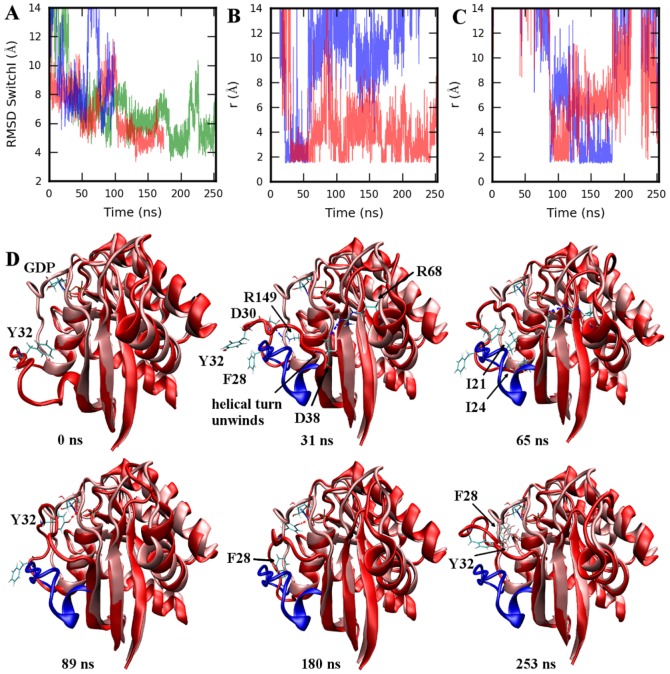
All-atom simulation of 4Q21-OpenSI at 360K. A. RMSD of SwitchI residues 25–40 (in 4Q21-OpenSI-Run1 (blue), Run2 (red), Run3 (green)) with respect to its conformation in 4Q21. B. Distance between the hydrogen bond donor/acceptor atoms for residue pairs D30-R149 (blue), D38-R68 (red), and in C. between Y32-P*_α_* group (blue) and Y32-ribose group (red). D. Time series plot of 4Q21-OpenSI-Run3. Simulated structure (red) is superimposed on 4Q21 (pink). Starting open conformation of SwitchI (residues 25–40) is also shown in blue for reference. SwitchI rises up to close the nucleotide pocket. In the final structure at the simulation end (253 ns) F28 can be seen in contact with GDP. Structure alignment was performed using backbone atoms N, CA, and C of core residues as defined in [18].

In agreement with the results from CG simulations, destabilization of GDP was observed in the all-atom simulation of 4Q21-OpenSI (more about it will be said in the next section); however, it occurred only in 1/3 simulations (4Q21-OpenSI-Run2) whereas GDP/Mg remained stable in the other two runs. As predicted by CG simulations, SwitchI did rise up towards GDP in all-atom simulations to close the nucleotide pocket (Figure 3) with RMSD of SwitchI (compared to its conformation in 4Q21) going below 4 Å (green in Figure 3A). Unwinding of the helical turn (formed by residues 37–39), an essential step for complete transition of open SwitchI towards its closed state, was observed in 2/3 all-atom simulations, while it remained intact in 4Q21-OpenSI-Run1 in which, as in CG simulation, the RMSD of SwitchI residues remained close to 7 Å (blue in Figure 3A). Transition of SwitchI towards its closed state was mediated by several polar and hydrophobic contacts formed between residues of SwitchI and the surrounding regions including 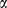
1 (residues 16–24), 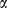
2, loop10 (residues 145–150, L10), and GDP itself.

Figure 3 summarizes the identified important events/interactions involved in complete SwitchI transition (observed in 4Q21-OpenSI-Run3). Polar contacts are mainly formed with the residues in L10 and 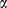
2. As observed in the CG trajectory, R149 and K147 forms salt bridge interactions with residues in the N-terminal part of SwitchI, mainly D30 and E31 (Figure 3B). Unwinding of the helical turn in the early stages of the simulation, allows E37 and D38 to form salt bridge interactions with R68 (Figure 3B), which induces residues 38–40 to position in close contact with 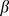
3 (residues 50–58) residues, eventually completing 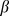
2 formation. As also previously observed in the 4Q21S39A simulation (Figure S6B), Y32 sidechain reorients towards GDP, mediated by hydrophobic interactions of Y32 with I21 and I24, and forms hydrogen bonds with P*_α_* and ribose group (Figure 3C) stabilizing an intermediate SwitchI conformation. The closed state of SwitchI is achieved at around 182ns when the F28 sidechain reorients and establishes contact with the base group causing a further change in the backbone conformation of SwitchI (RMSD goes down to around 4 Å), breaking GDP-Y32 sidechain interactions.

### Weak interaction at the base binding region destabilizes GDP: The role of D119

The CG simulation of 4Q21-OpenSI showed that weak Mg/base binding interaction led to destabilization of GDP in the nucleotide binding pocket. We validate the results of CG simulations with all-atom simulations of 4Q21 and 4Q21-OpenSI. In 4Q21, the base group forms hydrogen bonds with the D119 sidechain. Compared to its position in 4Q21, the C*_α_* atom of D119 is displaced by about 4.1 Å in the HRAS-GEF crystal structure [7]. SMD simulation of 4Q21-OpenSI was performed to assess the importance of base-D119 interaction in GDP stabilization.

**Figure 4:**
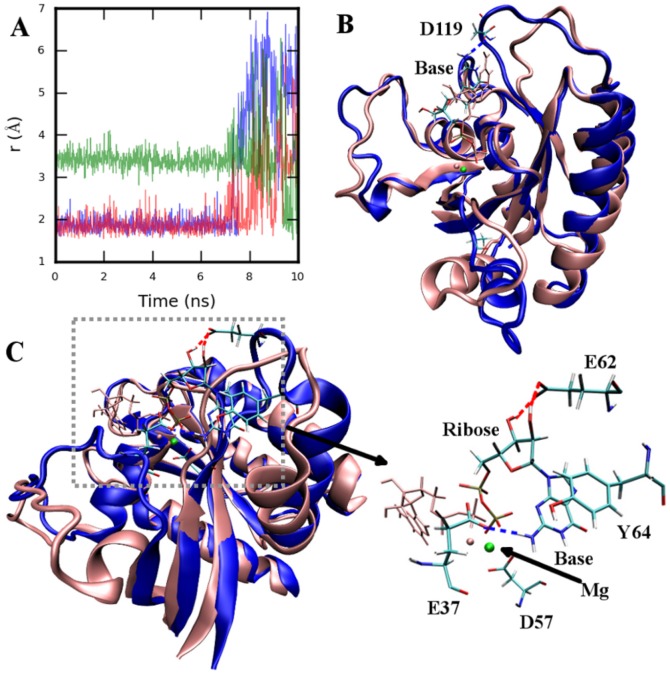
All-atom SMD and CMD of 4Q21-OpenSI at 360K. A. Distance between atom pairs D119OD1-baseH1 (blue), D119OD2-baseH21 (red), D119OD2-baseH22 (green). B. Structure at the end of SMD simulation (blue) superimposed on top of starting structure (pink). Base is displaced from its starting position (GDP drawn in pink) as D119 is pulled. C. Structure at the end of the CMD simulation (blue) continued from the last snapshot of SMD. Starting position of GDP/Mg is shown in pink. Both GDP/Mg is destabilized and displaced away from the nucleotide pocket. E37, D57, E62, and Y64 are shown stabilizing the leaving GDP/Mg. Structure alignment was performed using backbone atoms N, CA, and C of core residues as defined in [18].

In the SMD simulation, the C*_α_* atom of D119 was pulled towards its position in the HRAS-GEF complex with a constant velocity of 1.2 Å/ns and a force constant of 7.175 kcal/mol/Å^2^. Displacement of D119 (and thereby L8 residues 118–125) caused increased fluctuations of the base group (RMSD of GDP in blue in Figure S9A) that resulted in the weakening of the base-D119 interactions (after 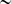
7ns in Figure 4A). Steering forces from the SMD simulations were removed and a CMD simulation was continued from the last snapshot of the SMD simulation (blue conformation in Figure 4B). Early in the CMD simulation, the transient contact between D119 and the base group was lost, resulting in destabilization of the base and ribose group (RMSD of GDP in red in Figure S9A). Enhanced fluctuations in the base and ribose group induced increased fluctuations in the phosphate groups (red in Figure S9B), weakening the phosphate-Ploop (residues 10–16) interactions. Loss of P*_β_*-Ploop contacts (Figure S9C) resulted in displacement of GDP away from the nucleotide binding pocket. The P*_β_* was displaced by 4–5 Å from its position in the starting structure (red in Figure S9B). The displaced nucleotide group then formed stabilizing interactions with the residues in L4 and C-terminal end of SwitchI. Figure 4C shows the conformation at the end of the simulation; E37 and L4 residues G60, Y64 stabilizes the base group while ribose forms hydrogen bond with E62. P*_β_* group forms interaction with K16. Interestingly, Mg remained bound to GDP and D57 throughout the simulation. A time-series plot of the SMD/CMD run is shown in Figure S9D.

In the previous simulation, the destabilization of the base interactions was initiated in the SMD simulation. Interestingly, similar events as seen in the above simulation were also observed in the CMD of 4Q21-OpenSI with D119A mutation (4Q21-OpenSI-D119A). In the absence of D119 interaction, the base group was displaced from its starting position after about first 14ns (RMSD of GDP in Figure S10A) resulting in increased fluctuations of GDP. This was followed by disruption of the Ploop-phosphate group contacts displacing P*_β_* by as much as 6–7 Å from its position in the starting conformation (Figure S10B). As before, the displaced GDP/Mg was stabilized by contacts with SwitchII loop and the C-terminal of SwitchI. Unlike previous simulation, GDP is seen in a different orientation (Figure S11A) and the base region is stabilized by interactions with E62 and G12 while the phosphate group forms interactions with G60, K16 (Figure S11B). Mg, as before, was surrounded by negatively charged residues, D57 and E37. Note, similar events were also observed in the destabilization of GDP in the 4Q21-OpenSI-Run2 simulation.

Simulation of 4Q21-D119A, a D119 mutant in closed SwitchI conformation, also resulted in destabilization of GDP (Figure S11C). The base region was displaced after about first 9ns although it remained in contact with F28. The base region destabilizes completely with the loss of F28 contact, after about 76ns. This was followed by breaking of ribose group-SwitchI contacts at around 95ns; phosphate-Ploop contacts remained intact within the simulation period.

Weakening of the Mg binding instead of the base in the 4Q21-OpenSI-S17AD57A simulation (4Q21-OpenSI with a double mutation, S17A and D57A), however, did not result in GDP destabilization within the simulation time. SwitchI transitions to its closed state as observed in the 4Q21-OpenSI simulations before, although with one difference. In the 4Q21-OpenSI-S17AD57A simulation, SwitchI rises up towards GDP in the first 40ns (Figure S12) with F28 folding inwards first, as opposed to Y32 in the 4Q21-OpenSI simulation, and establishing contact with the base while D33 sidechain mainly interacted with the ribose group and only transient hydrogen bonds were formed between Y32 backbone and ribose group.

## Simulation of Y32 mutant

Our simulations of 4Q21S39A and 4Q21-OpenSI showed that Y32 is among the crucial players in mediating the transition of SwitchI from open to closed state and vice versa. To further understand its role in SwitchI transitions all-atom simulations of 4Q21-OpenSI-Y32A mutant were performed. While in the 4Q21-OpenSI-Y32A-Run2 SwitchI transitioned to its closed state (Figure S13A-B); an entirely different conformation of SwitchI was seen in 4Q21-OpenSI-Y32A-Run1 compared to any of the previous 4Q21-OpenSI (wild type and mutant) simulations. As shown in Figure S13C, SwitchI did not rise up towards GDP; contrarily, it opened up even further with its RMSD reaching upto 20 Å compared to 4Q21 (red in Figure S13D). Also, within the simulation at around 80ns, the base-D119 contact was lost destabilizing GDP (Figure S13C). Further opening of SwitchI for prolonged period was not observed in the Y32F mutant simulations (Figure S14).

## Major players in SwitchI transition from open to closed state

**Figure 5:**
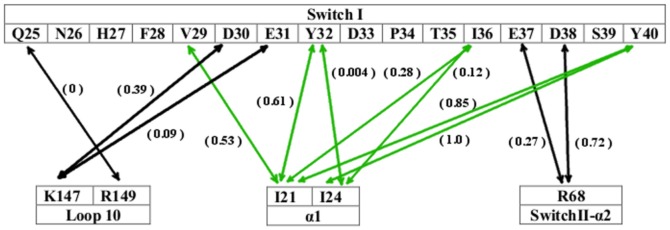
Common contacts identified in different 4Q21-OpenSI simulations that helps SwitchI rise up towards GDP from its open conformation. Black arrows represent polar contacts (both electrostatic and hydrogen bond) while hydrophobic contacts are shown by green arrows. Numbers in the bracket provides a rough estimate of the strength of each contact.

We identified only the common contacts between the SwitchI residues and the surrounding regions as seen in different 4Q21-OpenSI simulations (Text S1 and Table S3 provides details). Hydrophobic contacts were formed with the residues in 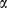
1, while the polar contacts formed mainly with the residues in L10 and 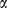
2 (Figure 5). I21, in particular, forms strong contacts with almost every other hydrophobic residue in SwitchI. F28-I21 contacts were formed mostly in the SwitchI conformations that were within 5 Å RMSD with its closed state. Importance of hydrophobic interactions, in particular of I21, in SwitchI transition was further verified in the simulations of 4Q21-OpenSI with I21 mutated to serine. Partial folding of SwitchI was observed in 4Q21-OpenSI-I21S-Run1 (blue in Figure S15A), with Y32 and S21 forming hydrogen bonds in the early stages of the simulation; reorientation of Y32 occurred, as seen in previous simulations, forming hydrogen bonds with phosphate and ribose group of GDP (Figure S15B), replacing the Y32-S21 contacts. Interestingly, in 4Q21-OpenSI-I21S-Run2, SwitchI failed to rise up towards GDP (RMSD in red in Figure S15A) and was seen in an entirely different conformation with residues of SwitchI forming interactions with residues in the 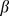
3 and SwitchII regions. For example, Y32 forms hydrogen bonds with D54 and hydrophobic contacts with L56 (Figure S15C, D).

## PCA of closed and open SwitchI states

**Figure 6:**
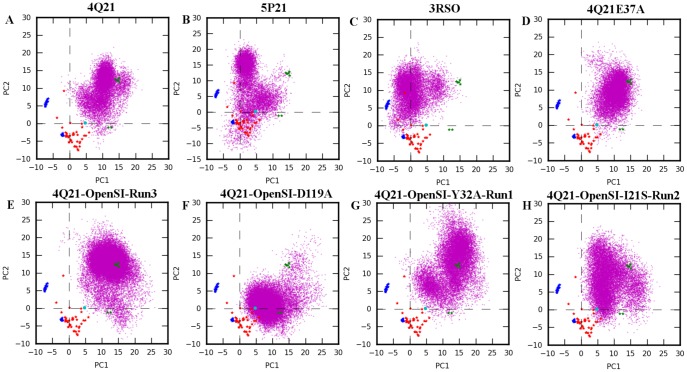
PCA of selected all atom simulations in closed and open SwitchI states. Trajectories were projected onto the first two PCs obtained using 71 structure dataset. Crystal structures are shown by colored stars where the coloring scheme is same as in Figure 1. Experimental structure of HRAS obtained from HRAS-GEF bound structure (PDB id: 1BKD) is also projected onto the subspace and is shown as cyan colored circle. MD conformers are shown as magenta dots. Panel A-D corresponds to simulations started in closed SwitchI state, namely: 4Q21 at 360K, 5P21 at 400K, 3RSO at 360 K, and 4Q21E37A at 360K. Panel E-H corresponds to simulations started in open SwitchI state performed at 360K, namely: 4Q21-OpenSI-Run3, 4Q21-OpenSI-D119A, 4Q21-OpenSI-Y32A-Run1, and 4Q21-OpenSI-I21S-Run2.

The conformational space sampled by different all atom simulations was evaluated by projecting the trajectories onto the subspace defined by the first two PCs obtained from the 71 structure dataset. Figure 6A-D shows the PCA of selected wild type and mutant structure simulations starting in closed SwitchI state at higher temperatures. On comparison with simulations performed at 300K (Figure S3), it is evident that significantly larger fluctuations occur in simulations at higher temperature. Both 4Q21 and 5P21 simulations sampled conformations in the vicinity of clusters 2 and cluster 1 respectively; however, these simulations did not sample region corresponding to cluster 3 (Figure 6A-B). Interestingly, simulations starting in cluster 3 (3RSO simulations) evolved towards structures of cluster 2 (3RSO simulation at 300K; magenta in Figure S3C) and cluster 1 (3RSO simulation at 360K; Figure 6C).

Figure 6E-H shows the PCA of selected simulations starting in open SwitchI state. Even though the 4Q21-OpenSI-Run3 simulation samples large space but the sampling is mostly limited to the region around cluster 1 indicating the transition from open to closed SwitchI state (Figure 6E). In the 4Q21-OpenSI-D119A simulation, as the GDP/Mg destabilizes and moves away from the nucleotide pocket, the two switch regions samples the subspace mainly around the conformation of HRAS as observed in the HRAS-GEF complex (PDB id: 1BKD; cyan circle Figure 6). Mutation of I21 or its interacting partner (Y32) led to enhanced fluctuations in switch regions which is evident as both the mutant simulations sampled a wide region of conformational space (Figure 6G-H).

## Discussion

From the PCA of 71 crystal structures of HRAS, we identified a new set of GTP-bound structures that form a separate cluster (cluster 3 in Figure 1; Figure S1) on the PC1-PC2 plane in addition to the two clusters identified previously by Gorfe et al. [18]. 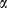
2 in cluster 3 structures occupies an intermediate position compared to the one observed in cluster 1 and cluster 2 structures (Figure 1C). Simulation of 3RSO (representative structure of cluster 3) at 300K identified interactions seen in both 5P21 and 4Q21 simulations. These observations along with the observation that 3RSO evolves from cluster 3 towards cluster 2 (Text S1; Figure S3C) and cluster 1 (Figure 6C) in the all-atom simulations, suggests that the structures of cluster 3 represent an intermediate conformation of HRAS (compared to 4Q21 and 5P21) with a low energy barrier for transition from cluster 3 to cluster 2 or cluster 1 but not the other way round as both 5P21 and 4Q21 simulations (even at high temperatures) did not sample the region corresponding to cluster 3 (Figure 6; Figure S3A-B).

Our CG and all-atom simulations demonstrate that the opening of SwitchI is an intrinsic motion of HRAS (occurs in both the GDP and GTP bound state; Figure S5) and although shown previously to occur in the Mg-free HRAS simulations [29], our results show that the open SwitchI conformations are accessible in the presence of Mg. Interestingly, the factors controlling the SwitchI conformations differed in the GDP- and GTP-bound states. Our simulations suggest that opening of SwitchI in the active state (GTP-bound) is a result of weak T35-Mg coordination (also reported in [15]) and a loss of T35-D57 hydrogen bond. In 4Q21 (GDP-bound) instead, coupling between the two switch regions due to E37-R68 interaction (also reported previously in [13], [15]) and the network of hydrogen bonds formed by residues S39, R41, D54, and Y71 was observed. Weakened coupling results in a faster opening of SwitchI lasting for an extended period; this was demonstrated in the simulations of mutant structures 4Q21E37A and 4Q21S39A (Figure 2) in which, unlike 4Q21 simulations, increased fluctuations also occurred in the N-terminal part of SwitchI (Figure S6, S7).

To understand the importance of a completely open SwitchI conformation in GDP release, we performed CG and all-atom simulations of 4Q21 starting in the open SwitchI state (4Q21-OpenSI). While destabilization of GDP was observed in only 1/3 4Q21-OpenSI all-atom simulations (4Q21-OpenSI-Run2), CG simulations resulted in frequent destabilization of GDP/Mg eventually displacing them from the nucleotide pocket. The parameters for CG GDP/Mg-protein interactions were determined by studying the GDP/Mg stability in 4Q21 (closed SwitchI state); their frequent destabilization thus points to some limitations of CG simulations. Interestingly, both CG and all-atom simulations resulted in open SwitchI folding back towards its closed state (Figure 3, Figure S8, S12, and S13A-B) indicating that the open conformation of SwitchI is an unstable state in the presence of bound nucleotide/Mg.

Several polar and hydrophobic contacts between SwitchI and residues in 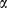
1, 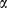
2, L10, and GDP were identified to mediate this transition. While simulations differed in specific interactions and the sequence of events leading to closed SwitchI state, certain contacts remained common among these simulations (Figure 5). Of these, hydrophobic contacts formed by I21 were found to be critical as I21S mutation interfered in the proper transition of open SwitchI back to its closed state (Figure S15C-D). Y32, on the other hand, not only forms polar contacts with GDP, but also forms strong hydrophobic contacts with I21. As seen in the Y32A mutant simulations, weakening of Y32-I21 hydrophobic interactions can result in even further opening of SwitchI (compared to its open state in HRAS-GEF crystal complex) delaying SwitchI closing, thereby giving ample time for GDP dissociation to occur (Figure S13C). By contrast, mutation of Y32 to a strong hydrophobic residue (Phe) did not result in further opening of SwitchI (Figure S14).

Disruption of base-D119 contact in all-atom simulations resulted in complete destabilization of GDP, displacing the P*_β_* atom (and thereby GDP) by at least 5–6 Å away from its starting position in the nucleotide binding pocket. The displaced GDP/Mg group was then stabilized by interaction with the residues in the two switch regions preventing its complete release from the protein within the simulation time. This is seen in the SMD/CMD run of 4Q21-OpenSI (Figure 4, Figure S9) in which the destabilization of the base was initiated by pulling D119 (thereby L8) towards its position in the HRAS-GEF crystal complex. Similar results were also obtained in the simulation of 4Q21-OpenSI-D119A mutant (Figure S10) establishing that the base destabilization (and eventually GDP) is initiated by the breaking of D119-base contact and not the steric interaction as a result of displacement of L8 (as seen in SMD simulation and HRAS-GEF crystal complex). Also, even in the 4Q21-OpenSI-Run2 and 4Q21-OpenSI-Y32A-Run1, GDP destabilization was initiated once the contact between the base-D119 was lost. Further, simulations of 4Q21 (closed SwitchI state) with D119A mutation also resulted in the destabilization of GDP (Figure S11C). Thus the loss of base-HRAS contact can destabilize GDP independently of the open/closed conformation of SwitchI. On the other hand, weakening of the Mg binding site (double mutant 4Q21-OpenSI-S17A-D57A) instead of base binding did not result in GDP destabilization with in the simulation time. Mutations of S17 and D57 are shown to increase the intrinsic nucleotide dissociation rate [30].

## Conclusions

A multiscale approach including CG-simulations, all-atom CMD and SMD simulations in combination with PCA was used to study wild type and mutant HRAS with the goal to identify structural features that determine the intrinsic nucleotide (GDP) exchange reaction, and use that knowledge to map out the specific roles of GEF in accelerating the exchange reaction. Molecular understanding of the process is essential, since recent efforts have focused on inhibiting the Ras-GEF interaction as a way to prevent Ras-driven tumors [31], [32].

PCA analysis of available HRAS crystal structures identified a new cluster of GTP-bound structures that have low barrier for transition towards clusters represented by 4Q21 (GDP-bound) and 5P21 (GTP-bound) structures. These structures thus provide an attractive starting conformation to study and understand the transition between the GDP-GTP bound states in wild type protein.

The open conformation of SwitchI, as seen in the HRAS-GEF crystal structure, is critical for GDP release [7]. Our results show that weakening the coupling between the two switch regions accelerates the opening of SwitchI in a mechanism that is different for different nucleotide binding conformations. Specifically, we note that residues S39, D54, and Y71 that contribute to the coupling in 4Q21, were recently identified to be a part of an allosteric pocket [33]; additionally, binding of small molecule in this region was shown to block GEF-mediated nucleotide release, but had no effect on the intrinsic exchange reaction in KRAS [31]. The binding of small molecule results in Y71 displacement which would disrupt the D54 mediated coupling. However, the small molecule itself interacts with S39 and T74 (among other interactions), and could couple the two switch regions [31]. We suggest that the binding of a small molecule did not disrupt the coupling between the two switch regions, and thus had no effect on the intrinsic exchange reaction. An open conformation of SwitchI is unstable in the absence of GEF, and rises up towards GDP to close the nucleotide pocket. As described in Figure 3 and Figure 5, the mechanism involves several polar and hydrophobic interactions.

I21 forms the core of the hydrophobic interactions responsible for SwitchI closing. Weakening of this core, either by mutation of I21 or its interacting partners (such as Y32), prevented or delayed the proper closing of an open SwitchI and is likely the reason for experimentally observed accelerated intrinsic nucleotide dissociation in Y32 mutants [12]. Interestingly, I21 forms part of a novel drug binding allosteric pocket [33]. Drug binding in this pocket was shown to prevent GEF-mediated exchange reaction possibly by stabilizing Ras in a conformation unsuitable for GEF-binding [32]. Our results show that inhibition of I21 alone can trigger alternative conformations of switch regions (Figure S15) which may affect GEF binding.

Loss of base-D119 contact resulted in the destabilization of GDP, independently of the open/closed state of SwitchI. This finding correlates well with the experimental studies demonstrating a strong decrease in the nucleotide affinity in D119 mutants [34]. Thus, an open conformation of SwitchI, as seen in the HRAS-GEF crystal structure, is not required for GDP destabilization and is mostly required for GDP release from HRAS. As discussed in Ref [17], in addition to SwitchI opening, a GEF-mediated exchange reaction involves rearrangement of L4 allowing A59 to interfere with Mg binding and E62 forms interaction with K16 weakening P*_β_*-Ploop interaction. Further, in the presence of GEF, dissociation of Mg may precede GDP release as indicated by the ternary complex of plant G-protein [17]. In contrast, no such rearrangement of SwitchII loop (L4) was observed in our simulations (in the absence of GEF). Mg remained bound to GDP and the GDP/Mg were displaced from the pocket together. Interestingly, the displaced GDP/Mg group in the simulations were stabilized by interactions with residues in the two switch regions (including E62 and K16), slowing down the release process. It is therefore possible that in the GEF mediated exchange Mg may dissociate first to accelerate the process in HRAS as well.

**Figure 7:**
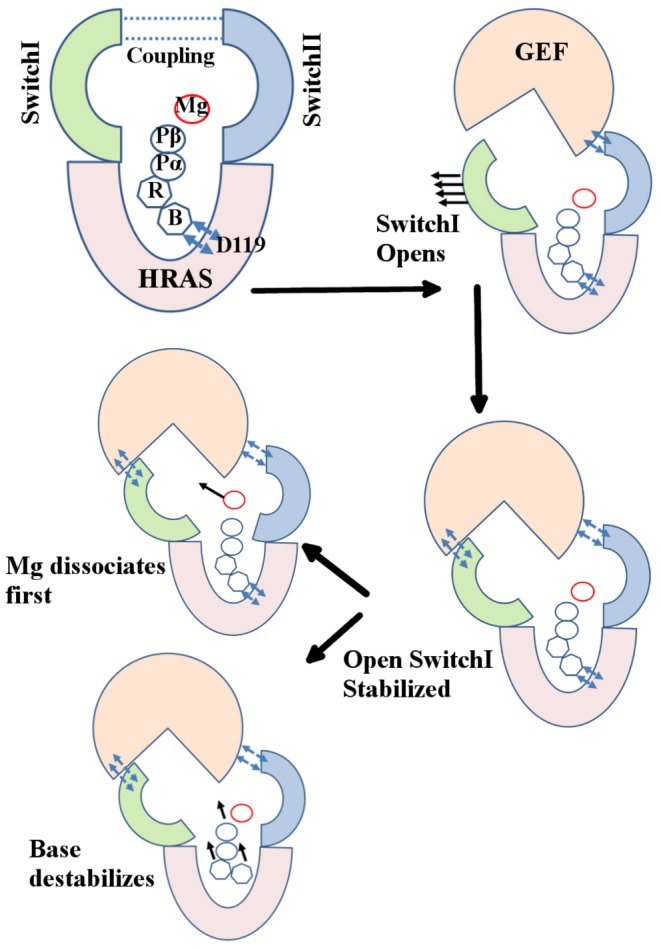
Schematic diagram of proposed mechanism of GEF action. Binding of GEF breaks the coupling between the SwitchI and SwitchII, resulting in an accelerated opening of SwitchI. The resulting open conformation of SwitchI must be stabilized by GEF to prevent its transition from open to closed state and thereby also ensuring that the leaving GDP/Mg is not stabilized by interactions with switch regions. Finally, unbinding of GDP may proceed either via breaking of base-D119 contact resulting in accelerated exit of GDP/Mg as a unit (as both the switch regions are engaged by GEF) or possibly a GEF-mediated rearrangement of L4 may allow Mg dissociation to precede GDP release.

Based on our results, a GEF mediated exchange reaction could be thought to proceed in the following manner (Figure 7). First, binding of an incoming GEF to the SwitchII region would break the coupling between the SwitchI and SwitchII, resulting in an accelerated opening of SwitchI. Second, the GEF must stabilize the open conformation of SwitchI to prevent its closing, and thereby also ensuring that the leaving GDP/Mg is not stabilized by interactions with switch regions. As seen in the HRAS-GEF crystal complex (PDB id: 1BKD), GEF not only forms extensive interactions with SwitchI stabilizing its open conformation but also engages almost every residue of SwitchII including Y64, R68, and Y71 which would not only break the coupling between the two regions but will also prevent their interaction with the leaving GDP/Mg group. Finally, unbinding of GDP may proceed via breaking of base-D119 contact resulting in accelerated exit of GDP/Mg as a unit (as both the switch regions are engaged by GEF) or possibly a GEF-mediated rearrangement of L4 (as seen in 1BKD) may allow Mg dissociation to precede GDP release. It is our expectation that the detailed predictions presented in this paper will trigger further experimental results.

## Supporting Information

Figure S1 **PCA analysis of experimental structures.** Projection of structures onto the first two PCs obtained using A) 46 structure dataset and B) 71 structure dataset. Green: HRAS-GDP bound; Red, Blue: HRAS-GTP bound conformations. Experimental structures groups in to three major clusters highlighted in B.
(EPS)

Figure S2 **RMSD and C** ***_α_*** **atom RMSF observed in CG and all-atom simulations of 4Q21 (A, B), 5P21 (C, D), and 3RSO (E, F).** Results are shown for three CG simulations (differing only in initial assignment of velocities; shown in blue, red, and green) where each run was performed for 100 million time-steps at T = 0.8. RMSF calculation was done for final 95 million time-steps. Black dotted line in B and D corresponds to RMSF calculated for first 45 million time-steps. All-atom simulation was performed at T = 300K. RMSF calculation for all-atom simulation (shown in magenta) was done for 99 ns production run.
(EPS)

Figure S3 **Projection of CG and all-atom simulations on to the first two PCs obtained from 71 structure dataset.** A. 4Q21. B. 5P21. C. 3RSO . Color codes are same as in Figure S2. Crystal structures are shown by colored stars where the coloring scheme is same as in Figure 1.
(EPS)

Figure S4 **Cross-correlation plot from CG and all-atom simulations.** Left panel (A, C, E) shows the plot from CG simulations performed at T = 0.8 while right panel (B, D, F) shows the plot from all-atom simulations run at T = 300K.
(EPS)

Figure S5
**C** ***_α_*** **-RMSF from 99 ns all-atom simulations of A. 4Q21, B. 5P21, C. 3RSO at temperatures 300K (blue), 360K (red), and 400K (green).** Also shown are the snapshot from higher temperature simulations (360K for 4Q21 and 3RSO and 400K for 5P21) that highlights the open SwitchI/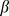
2 conformation. Simulated structure is shown in blue superimposed on respective experimental structure shown in pink. Structure alignment was performed using backbone atoms N, CA, and C of core residues as defined in [18].(EPS)

Figure S6
**All-atom simulation of mutant 4Q21S39A and 4Q21E37A.** A. Snapshot of 4Q21S39A (green), and 4Q21E37A (red) showing open SwitchI/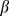
2 state superimposed on top of native state in pink. B. Snapshot of 4Q21S39A (green) in open SwitchI/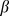
2 conformation highlighting hydrogen bond between Y32 and oxygen atom of phosphate group on GDP. Structure alignment was performed using backbone atoms N, CA, and C of core residues as defined in [18].(EPS)

Figure S7 **Histogram of distances between C** ***_α_*** **atom of selected SwitchI residues and representative atoms on GDP (N9 of base and P** ***_β_*** **) from four different all-atom simulations: 4Q21 run at 300K (pink), 4Q21 run at 360K (blue), 4Q21E37A run at 360K (red), and 4Q21S39A run at 360K (green).** Bin width was set to 0.1 Å. All four simulations have equal number of data points.
(EPS)

Figure S8 **Coarse-grained simulation of 4Q21-OpenSI.** A. 4Q21-OpenSI simulated structure at the start of the simulation (red), at the end of the simulation (blue) superimposed on 4Q21 (pink). SwitchI rises up towards GDP. GDP/Mg destabilizes and are displaced from the nucleotide pocket. Coarse grained GDP beads are shown as spheres with base (orange), ribose (yellow), PA and PB (cyan) and Mg (green). B. Sidechain hydrogen bonds formed during the simulation between residue pairs D30-R149 (blue), H27-E153 (red), T20-T35 (green), D30-K147 (black), and sidechain-mainchain hydrogen bond between S17-Y32 (magenta). C. RMSD of SwitchI residues 25–40 (in 4Q21-OpenSI) with respect to its conformation in 4Q21. Structure alignment was performed using backbone atoms N, CA, and C of core residues as defined in [18].
(EPS)

Figure S9 **Combined all-atom SMD/CMD trajectory of 4Q21-OpenSI at 360K.** A. RMSD of GDP (with respect to its position in starting structure) in SMD simulation (blue) and CMD simulation (red). RMSD of GDP in 4Q21OpenSI-Run3 (magenta) is shown for reference. B. Distance of P*_β_* in simulated structure from its position in starting structure. Color codes are same as in A. C. Distance between atoms pairs K16HZ2-GDPO1B (blue), S17HG1-GDPO3B (red), and G15HN-GDPO1B (green). D. Time series plot of SMD/CMD simulation. Residues E37, D57, E62, Y64, GDP, and Mg (green sphere) are highlighted in simulated structure. GDP and Mg in starting conformation is shown in pink.
(EPS)

Figure S10 **All-atom CMD simulation of 4Q21-OpenSI-D119A at 360K.** A. RMSD of GDP with respect to its position in starting structure. B. Distance of P*_β_* in simulated structure from its position in starting structure.
(EPS)

Figure S11 **All atom simulation of 4Q21-OpenSI-D119A and 4Q21-D119A at 360K.** A. Structure of 4Q21-OpenSI-D119A at the end of the CMD simulation (blue, drawn in transparent) superimposed on top of starting structure (pink, drawn in transparent). GDP/Mg destabilizes and is displaced away from its starting position (GDP drawn in bond representation in pink, Mg as pink sphere) where it coordinates with E62, K16, G12, G60. Mg is trapped between E37 and D57. B. Distance between atoms pairs E62OE2-GDPH22 (blue), G12HN-GDPN7 (red), G60HN-GDPO2A (green), and K16HZ1-GDPO2A (black). C. Snapshots from simulation of 4Q21-D119A at 10, 80, and 100ns. F28 is highlighted to show interaction with base group at 10ns. Structure alignment was performed using backbone atoms N, CA, and C of core residues as defined in [18].
(EPS)

Figure S12 **All atom simulation of 4Q21-OpenSI-S17AD57A at 360K.** A. RMSD of SwitchI residues 25–40 with respect to its conformation in 4Q21. B. Conformation at the end of the simulation. Simulated structure (red) is superimposed on 4Q21 (pink). Starting open conformation of SwitchI (residues 25–40) is also shown in blue for reference. SwitchI rises up to close the nucleotide pocket. F28 and GDP is also shown in the simulated structure.
(EPS)

Figure S13 **All atom simulation of 4Q21-OpenSI-Y32A at 360K.** A. RMSD of SwitchI residues 25–40 (in 4Q21-OpenSI-Y32A-Run2) with respect to its conformation in 4Q21. B. Conformation at the end of the 4Q21-OpenSI-Y32A-Run2 simulation. Simulated structure (red) is superimposed on 4Q21 (pink). Starting open conformation of SwitchI (residues 25–40) is also shown in blue for reference. C. Conformation at 100ns in the 4Q21-OpenSI-Y32A-Run1 simulation. SwitchI opens up even further in this simulation. Base group of GDP loses contact with D119 after about 80ns. D. RMSD of SwitchI residues 25–40 (in 4Q21-OpenSI-Y32A-Run1) with respect to its conformation in starting structure (blue) and 4Q21 (Red).
(EPS)

Figure S14 **All atom simulation of 4Q21-OpenSI-Y32F at 360K.** A. RMSD of SwitchI residues 25–40 in 4Q21-OpenSI-Y32F-Run1 (blue) and Run2 (red) with respect to its conformation in 4Q21. B. Conformation at the end of the 4Q21-OpenSI-Y32F-Run1 (blue) and Run2 (red). The starting conformation is shown in pink.
(EPS)

Figure S15 **All atom simulation of 4Q21-OpenSI-I21S at 360K.** A. RMSD of SwitchI residues 25–40 in 4Q21-OpenSI-I21S-Run1 (blue) and 4Q21-OpenSI-I21S-Run2 (red) with respect to its conformation in 4Q21. B. Conformation at the end of the 4Q21-OpenSI-I21S-Run1 simulation. Simulated structure (red) is superimposed on 4Q21 (pink). Starting open conformation of SwitchI (residues 25–40) is also shown in blue for reference. Y32 can be seen making hydrogen bonds (red dashed line) with the phosphate group of GDP. C. Conformation at the end of the 4Q21-OpenSI-I21S-Run2 simulation. Y32 forms hydrogen bonds with D54 and hydrophobic contact with L56. D. Side view of the conformation shown in C.
(EPS)

Table S1 **List of all-atom simulations performed.**
(PDF)

Table S2 **PDB id of 25 structures added to the 46 structure dataset used for PCA.**
(PDF)

Table S3 **List of representative sidechain atoms of hydrophobic and charged residues used for contact analysis.**
(PDF)

Text S1 **Supporting Information text.**
(PDF)
