# Supplementary material for: Mechanism of the Exchange Reaction in HRAS from Multiscale Modeling
Source: PLoS One. 2014 Oct 1;9(10):e108846. doi: 10.1371/journal.pone.0108846 (PMC4182752; doi:10.1371/journal.pone.0108846)
Supplement: Table S1 — List of all-atom simulations performed. (PDF) [file pone.0108846.s016.pdf]

Table S1: List of all-atom simulations performed.

PDB ID/Name	Temperature (K)	Equilibration (ns)	Production (ns)	Simulation Time (ns)
4Q21	300	1	99	100
4Q21	360	1	99	100
4Q21	400	1	99	100
5P21	300	1	99	100
5P21	360	1	99	100
5P21	400	1	99	100
3RSO	300	1	99	100
3RSO	360	1	99	100
4Q21E37A	360	1	99	100
4Q21S39A	360	1	99	100
4Q21-OpenSI-Run1	360	1	99	100
4Q21-OpenSI-Run2	360	1	174	175
4Q21-OpenSI-Run3	360	1	253	254
4Q21-OpenSI-S17AD57A	360	1	180	181
4Q21-OpenSI-SMD-CMD	360	1	104	105
4Q21-OpenSI-D119A	360	1	193	194
4Q21-D119A	360	1	100	101
4Q21-OpenSI-Y32A-Run1	360	1	284	285
4Q21-OpenSI-Y32A-Run2	360	1	174	175
4Q21-OpenSI-Y32F-Run1	360	1	99	100
4Q21-OpenSI-Y32F-Run2	360	1	99	100
4Q21-OpenSI-I21S-Run1	360	1	125	126
4Q21-OpenSI-I21S-Run2	360	1	125	126
