# Supplementary material for: Mechanism of the Exchange Reaction in HRAS from Multiscale Modeling
Source: PLoS One. 2014 Oct 1;9(10):e108846. doi: 10.1371/journal.pone.0108846 (PMC4182752; doi:10.1371/journal.pone.0108846)
Supplement: Table S2 — PDB id of 25 structures added to the 46 structure dataset used for PCA. (PDF) [file pone.0108846.s017.pdf]

Table S2: PDB id of 25 structures added to the 46 structure dataset used for PCA.

PDB ID	PDB ID	PDB ID	PDB ID	PDB ID
2RGA	2RGB	2RGC	2RGD	3K8Y
3LBH	3LBI	3LBN	3RRY	3RRZ
3RS0	3RS2	3RS3	3RS5	3RSO
3V4F	4DLR	4DLS	4DLT	4DLU
4DLV	4DLW	4DLX	4DLY	4DLZ
