# Supplementary material for: Mechanism of the Exchange Reaction in HRAS from Multiscale Modeling
Source: PLoS One. 2014 Oct 1;9(10):e108846. doi: 10.1371/journal.pone.0108846 (PMC4182752; doi:10.1371/journal.pone.0108846)
Supplement: Table S3 — List of representative sidechain atoms of hydrophobic and charged residues used for contact analysis. (PDF) [file pone.0108846.s018.pdf]

Table S3: List of representative sidechain atoms of hydrophobic and charged residues used for contact analysis.

Residue Name	Representative Atom
ALA	CB
ARG	CZ
ASP	CG
GLU	CD
ILE	CG1
LEU	CG
LYS	CE
MET	SD
PHE	CG
TYR	CG
VAL	CB
