# Supplementary material for: Mechanism of the Exchange Reaction in HRAS from Multiscale Modeling
Source: PLoS One. 2014 Oct 1;9(10):e108846. doi: 10.1371/journal.pone.0108846 (PMC4182752; doi:10.1371/journal.pone.0108846)
Supplement: Text S1 — Supporting Information text. (PDF) [file pone.0108846.s019.pdf]

Supporting Material for Mechanism of the exchange reaction in HRAS from multiscale modeling

Abhijeet Kapoor and Alex Travesset
Physics and Astronomy, Iowa State University, Ames, IA, U.S.A.

Supporting Methods

Coarse graining of guanine diphosphate/triphosphate

GDP/GTP was modeled as a four-/five-bead structure connected by harmonic bonds with each of the four/five groups, namely, base (B), ribose (S), α-, β-, and γ-phosphate (PA, PB, and PG) represented by one bead each. B and S beads were positioned at the center of mass of the respective rings. GDP/GTP was constrained by adding harmonic angles between the beads B-S-PA, S-PA-PB, and PA-PB-PG, equilibrium angle value for the same were obtained from all-atom simulation of GDP/GTP. Dihedral constraints between the beads B-S-PA-PB and S-PA-PB-PG were also added. Magnesium (Mg) was modeled as a single bead.

Two sets of CG simulations were performed. In the first set, the interactions of GDP/GTP and Mg with HRAS was modeled by connecting harmonic springs between the residue beads and GDP/GTP/Mg beads. These interactions follow the original experimental results in Ref. [1]. Overlap between the GDP/GTP and Mg beads with the protein was prevented by adding a purely repulsive 12-6 Lennard-Jones (LJ) potential. For S and B beads σ of 5Å was used for the interaction with protein sidechain beads while for rest of the interactions a size of 3.1Å was used for B, S, PA, PB and Mg beads to define σ with other protein atoms. Defining GDP/GTP-protein interactions as bonded limits the simulation to the study of equilibrium dynamics only. To model the nucleotide exchange process the bonded interactions (between GDP/GTP, Mg, and protein) were replaced with non-bonded potentials in the second set of simulations. A 12-6 LJ potential was used to define interactions between GDP/GTP, Mg, and protein beads. Bead sizes (for GDP/GTP and Mg) and interaction strengths (ϵ) were tuned such that the GDP/GTP and Mg was stabilized in the nucleotide pocket (in 4Q21 and 5P21 CG simulations). In the final parameter set, a σ of 4.5Å was used for the B and S beads interaction with protein sidechain and a radii of 2.5Å was used to define σ with backbone beads. For PA, PB, PG, and Mg beads σ of 3.85Å was used for interaction with protein sidechain beads while a radii of 2.3Å was used to define σ with the backbone beads. A σ of 3.1Å was used to define interactions between Mg and (PA, PB, PG) beads. Mg-(PB, PG) interaction strength was set to 13. Interaction strength between GDP/GTP/Mg and protein beads was set to 3.0.

Supporting Text

Equilibrium dynamics of HRAS from CG simulations are in good agreement with all-atom simulations

The overall native state of HRAS, both in the GDP- and GTP-bound state (with the interactions between nucleotide and protein modeled as harmonic springs), simulated with our CG model [2, 3] was stable with an average RMSD of about 3.7Å (for HRAS-GDP) and about 2.6Å (for HRAS-GTP). Large fluctuations confined mainly to the unstructured part of the protein involving residues in the two switch regions, SwitchI and SwitchII loop (L4), loops L7 (residues 105-109) and L8 (residues 118-125). However, certain parts of the structure that remained stable in the all-atom simulations showed large fluctuations within CG simulations. These fluctuations involved change in the orientation of helix $\alpha 4$ and unstable first four residues of N-terminal strand $\beta 1$, arises due to missing interactions as a result of one bead approximation of sidechain atoms (as discussed in Ref. [3]). We stabilize $\alpha 4$ by adding harmonic springs corresponding to missing sidechain hydrogen bond interactions, identified by comparison with all-atom simulations of both 4Q21 and 3RSO, between residue pairs R123-E143, R123-S127, S127-E143, Y141-E143, and mainchain hydrogen bond between L113-P140. Similarly, $\beta 1$ is stabilized by adding harmonic spring corresponding to unstable mainchain hydrogen bond for residues 2-4. An additional spring between residues Q22 and A146 is added corresponding to missing sidechain-mainchain hydrogen bond. These springs were added for all the subsequent CG simulations of HRAS discussed in the paper.

Figure S2 shows the time evolution of the RMSD with respect to the starting structure for both GDP-bound (4Q21; Figure S2A) and GTP-bound (5P21, Figure S2C; 3RSO, Figure S2E) HRAS from CG simulations (100 million timesteps; colored blue, red, and green) and all-atom simulation (99ns; colored magenta). Figure S2B, D, F shows the RMSF of the C_α atoms. In CG simulations, HRAS remains close to the starting structure (with an average RMSD of ≈(3.1, 4.0, 3.3) Å for three simulations of 4Q21 and ≈(2.3, 2.4, and 2.3)Å for 5P21) with fluctuations mainly seen in the different loop regions. SwitchI fluctuations are higher in the GDP-bound state than in the GTP-state due to the absence of SwitchI coordination with GDP and Mg. Overall, the fluctuations from the CG simulations are in good-agreement with those of the all-atom simulation (colored magenta), although comparatively higher fluctuations are seen for some of the residues especially in the region L6 (residues 85-86), L7, L8, L10 (residues 145-150), and $\alpha 3$. The disagreement in these regions plausibly results from enhanced sampling in CG simulations as RMSF from smaller time length CG trajectory (first 45 million timestep shown by black dotted line corresponding to Run1 for 4Q21 and Run2 for 5P21 in Figure S2B, D) shows reasonably better agreement with all-atom simulations for these regions.

The conformational space sampled by each CG and all-atom simulations was compared by projecting the trajectories on the first two PCs obtained from the 71 structure dataset (Figure S3). Both 4Q21 (except run2 in red) and 5P21 simulations sampled conformations in close vicinity of the starting structures with somewhat enhanced sampling in CG simulations, specially in the case of 5P21, where one of the simulation (run2 in red) sampled conformations corresponding to the inactive GDP-bound G12V mutant (PDB ID: 1Q21, 2Q21; 2 green stars). A distinct orientation of $\alpha 2$ occurs after partial opening of $\beta 2$ (residues 38-40), similar to the one observed in HRAS-GEF crystal complex [4], at the beginning of run2 in 4Q21. This results in the trajectory forming a separate cluster away from the starting structure (red in Figure S3A and also higher RMSD as seen in Figure S2A) on the plane defined by the first two PCs. In contrast, in the all-atom and CG simulations of 3RSO the simulated structure moves away from the third cluster formed by the GTP-bound conformations. While the overall structure remains close to the native state,

the difference between the CG and all-atom trajectory mainly comes from SwitchII, $\alpha3$, and L7. Within CG simulations, the initial structure undergoes rearrangements involving the opening of a helical turn formed by residues 62-64, which is then followed by a change in the orientation of the C-terminal of $\alpha3$ and L7 towards $\alpha4$ and subsequently, $\alpha2$ and L4 orients towards $\alpha3$. Within all-atom simulations, the helical turn opens but, unlike CG simulations, both $\alpha2$ and $\alpha3$ remained close to the starting conformations. The conformation of L4 changes, such that it first maps between the three clusters on the PC1-PC2 plane, and then by the end of the simulation, moves towards the conformation observed in the structures of cluster2 (magenta in Figure S3C).

The enhanced sampling achieved in CG simulations is further demonstrated by the cross-correlation plot (Figure S4), where the off-diagonal peaks observed in the CG simulations (left panel Figure S4) and the all-atom accelerated molecular dynamics (aMD) of HRAS [5] match well. Those correlations are largely absent in all-atom CMD simulations (right panel Figure S4). The most distinct part of the plot, not seen in aMD simulations of HRAS [5], is the correlation seen between region L6-$\alpha3$-L7 (residues 85-109) and $\alpha4$ (residues 127-137; left panel Figure S4). The regions $\alpha3$-L7-$\alpha4$ were recently shown to be involved in an allosteric binding with calcium acetate that led to ordered SwitchII placing Q61 in its precatalytic conformation [6]. Notice that although the off-diagonal peaks corresponding to the region L6-$\alpha3$-L7 and $\alpha4$ are seen in both GDP- and GTP bound CG simulations, the communication between SwitchII-$\alpha3$ is largely absent in the GDP bound state indicating that the allosteric switch formed by $\alpha3$-L7-$\alpha4$ will be active only in the GTP-bound state.

Determination of common contacts in SwitchI transition from open to closed state

Three different trajectories (4Q21-OpenSI-Run3, 4Q21-OpenSI-S17AD57A, and 4Q21-OpenSI-Y32A-Run2) were selected, for the identification of common contacts, based on the criteria that the GDP remained stable throughout the simulation and the RMSD of the SwitchI residues reached at least 4Å with respect to its conformation in 4Q21. In each of the simulations, trajectory frames starting from the beginning till the part when RMSD of the SwitchI reaches 5Å or below consistently within the simulation was used for the analysis. This was done to separate the contacts responsible for SwitchI rising from the one that forms to stabilize the already attained closed conformation. Two sidechains were considered to be in contact (either hydrophobic or electrostatic) if the distance between the representative atoms on the sidechains was less than 8Å. Hydrophobic contact was calculated between non-polar residues and electrostatic contact was calculated between charged residues Arg, Lys, Glu, and Asp. List of representative atoms for each amino acid sidechain is given in Table S3. Hydrogen bond contacts were identified using VMD. A contact was labeled as common if it formed in at least 2/3 simulations and occurred in more than 10% of the trajectory frames in each of the simulations. The strength of the contact was calculated by taking the ratio of the total number of frames from the three simulations in which the contact forms divided by the total number of frames from the three simulations used for analysis. The resulting values were normalized between 0 and 1 such that the strongest contact was assigned a value of 1 and the weakest 0.

References

1. Milburn M, Tong L, deVos A, Brunger A, Yamaizumi Z, et al. (1990) Molecular switch for signal transduction: structural differences between active and inactive forms of protooncogenic ras proteins. Science 247: 939-945.

2. Kapoor A, Travesset A (2013) Folding and stability of helical bundle proteins from coarse-grained models. Proteins 81: 1200–1211.

3. Kapoor A, Travesset A (2014) Folding 19 proteins to their native state and stability of large proteins from a coarse-grained model. Proteins 82: 505–516.

4. Boriack-Sjodin PA, Margarit SM, Bar-Sagi D, Kuriyan J (1998) The structural basis of the activation of ras by sos. Nature 394: 337-343.

5. Grant BJ, Gorfe AA, McCammon JA (2009) Ras conformational switching: Simulating nucleotide-dependent conformational transitions with accelerated molecular dynamics. PLoS Comput Biol 5: e1000325.

6. Buhrman G, Holzapfel G, Fetics S, Mattos C (2010) Allosteric modulation of ras positions q61 for a direct role in catalysis. Proc Natl Acad Sci USA 107: 4931-6.
